# Quantum Distance Measures Based upon Classical Symmetric Csiszár Divergences

**DOI:** 10.3390/e25060912

**Published:** 2023-06-08

**Authors:** Diego G. Bussandri, Tristán M. Osán

**Affiliations:** 1 Instituto de Física La Plata (IFLP), Consejo Nacional de Investigaciones Científicas y Técnicas de la República Argentina (CONICET), Diagonal 113 e/63 y 64, La Plata B1900, Argentina; 2 Instituto de Física Enrique Gaviola (IFEG), Consejo Nacional de Investigaciones Científicas y Técnicas de la República Argentina (CONICET), Av. Medina Allende s/n, Córdoba X5000HUA, Argentina; 3 Facultad de Matemática, Astronomía, Física y Computación, Universidad Nacional de Córdoba, Av. Medina Allende s/n, Ciudad Universitaria, Córdoba X5000HUA, Argentina

**Keywords:** Csiszár divergences, quantum metrics, distinguishability, Jensen–Shannon divergence, Hellinger distance, trace distance, triangular discrimination

## Abstract

We introduce a new family of quantum distances based on symmetric Csiszár divergences, a class of distinguishability measures that encompass the main dissimilarity measures between probability distributions. We prove that these quantum distances can be obtained by optimizing over a set of quantum measurements followed by a purification process. Specifically, we address in the first place the case of distinguishing pure quantum states, solving an optimization of the symmetric Csiszár divergences over von Neumann measurements. In the second place, by making use of the concept of purification of quantum states, we arrive at a new set of distinguishability measures, which we call *extended quantum Csiszár distances*. In addition, as it has been demonstrated that a purification process can be physically implemented, the proposed distinguishability measures for quantum states could be endowed with an operational interpretation. Finally, by taking advantage of a well-known result for classical Csiszár divergences, we show how to build quantum Csiszár true distances. Thus, our main contribution is the development and analysis of a method for obtaining quantum distances satisfying the triangle inequality in the space of quantum states for Hilbert spaces of arbitrary dimension.

## 1. Introduction

Quantum information theory aims to understand the fundamental principles regarding the behavior of quantum systems and how they can be useful in the search for new technologies. One of the central tasks in quantum information theory is to quantify the difference between two quantum states—quantum mechanics allows for superpositions of states, which can make it challenging to define proper notions of distance in the space of quantum states [1]. In this paper, we present a novel set of distinguishability measures for quantum states, termed *Quantum Csiszár divergences*. We demonstrate that these measures can be obtained through the maximization of classical Csiszár divergences over a set of von Neumann measurements performed on the quantum system.

Csiszár divergences, also known as *f*-divergences, are a widely used family of divergence measures between two probability distributions, with applications in a variety of fields, including classical statistics and information theory, among others [2]. This set includes a number of well-known divergences, such as the Jensen–Shannon divergence, the Kulback–Liebler divergence, the total variation distance, the triangular discrimination, and the Hellinger distance [3,4]. In addition, many *f*-divergences have interesting statistical and physical interpretations [5].

In the context of quantum information theory, in recent years, Csiszár divergences have gained significant attention as a tool for quantifying the difference between two quantum states. There has been a considerable amount of research in this direction. These new divergences have been shown to be related to a wide range of fundamental concepts in quantum information theory, such as entanglement theory, quantum error correction, and quantum channel capacities [1,6,7,8,9]. In general terms, the approach employed in the related literature is to introduce functionals depending on a defining Csiszár function f(u) defined over a set of operators. These functionals are often defined as convex optimization problems over a set of operators.

In this work, we present a different approach to link Csiszár divergences with the quantum realm. We introduce the corresponding Csiszár extensions showing that they are ultimately based on a measurement procedure. In this way, they form a set of distinguishability measures between quantum states [10,11]. Specifically, this article can be seen as an extension of Ref. [12], in which a method was developed to obtain a distinguishability measure in the space of quantum states, starting from the classical Jensen–Shannon divergence. Our findings lead to quantum distances based on any symmetric Csiszár divergence. Moreover, by means of a well-known result for classical Csiszár divergences, we show how to build quantum Csiszár distances fulfilling the triangle inequality. In this way, we are able to obtain true distances (often called *metrics*) in the space of quantum states. Even though from a mathematical viewpoint it is apparent that the feature of being a true distance is a basic requirement for a suitable distance measure, true distances in the quantum realm (i.e., distances in the space of quantum states that also satisfy the triangle inequality) are interesting in their own right as they find applications in a number of subjects of research related to quantum information theory and quantum information processing, such as measures of quantum correlations (including entanglement) and the evaluation of the convergence of iterative algorithms in quantum information processing, among others [13,14,15]. In addition, the triangle inequality plays a significant role in a variety of topics related to quantum information theory. Particularly, it allows one to improve converse bounds in channel discrimination theory [16], to establish entropic bounds in the study of non-Markovianity and information backflow in open quantum systems [17,18], to test the presence of quantum entanglement [19], and to define new distance measures in the space of quantum channels [20], among other uses [21,22,23]. This is an important step towards the development of a robust and efficient generalized theory about quantum distance measures, which has numerous applications in quantum communication, quantum computing, and quantum cryptography.

This work is organized as follows. The main theoretical framework is in the following Section. The *quantum Csiszár distances*, along with their extended version, are introduced and physically motivated in Section 3. In Section 3.1, it is established how to attain quantum true distances from symmetric Csiszár distances. The relevant Csiszár divergences are particularized in Section 3.2: the variational distance, Hellinger and triangular discrimination, and the Jensen–Shannon divergence are considered. This Section also includes (cf. Section 3.2.5) a comparison between the extended quantum Csiszár distances between quantum mixed states and the one obtained from the optimization over arbitrary von Neumann measurements. Final remarks are in Section 4.

## 2. Preliminaries

We introduce the main notation we use in this work. Let $\bar{R}=\left(-\infty ,\infty \right)$, $R_{+}=\left[0,\infty \right)$, and $R_{0}=\left(0,\infty \right)$. Regarding distinguishability quantifiers, we employ the following terms.

A *metric space* comprises an ordered pair (χ,d), where χ represents a set and $d:\chi \times \chi \to R_{+}$ is a real-valued function on the set χ, such that for any x,y,z∈χ, the following properties hold:

1. *Non-negativity*: d(x,y)≥0,
2. *Identity of indiscernibles*: d(x,y)=0 if and only if x=y,
3. *Symmetry*: d(x,y)=d(y,x),
4. *Triangle inequality*: d(x,y)≤d(x,z)+d(z,y).

χ may represent the set of probability distributions or quantum states. In addition, if a distance measure *d* only satisfies property 1, we call it a *divergence*, while if *d* satisfies the properties 1 to 3, we use the word *distance* [4,24].

### 2.1. Csiszár Divergences

A Csiszár divergence (also known as an *f*-divergence) between the probability distributions $P=\{p_{1},p_{2},\cdots ,p_{n}\}$ and $Q=\{q_{1},q_{2},\cdots ,q_{n}\}$ is defined as [2,3,4,14,25,26,27],

(1) $$D_{f}\left(P,Q\right)=\sum_{i=1}^{n}q_{i}\;f\;\left(\frac{p_{i}}{q_{i}}\right),$$

where $f:R_{+}\mapsto \bar{R}$ belongs to the set F of convex functions that are finite on $R_{0}$ and continuous on $R_{+}$. The set of Csiszár divergences might involve divergences, distances, and true distances (i.e., distances that also satisfy the triangle inequality 4) in the space of probability distributions. In Section 3.2, we consider particular cases of symmetric Csiszár divergences. Now, we introduce the main results of the properties of the Csiszár function f(u) and the quantity $D_{f}\left(P,Q\right)$.

#### 2.1.1. Basic Properties of Csiszár Divergences

Let $f^{*}\in F$, the *–conjugate (convex) function of *f*, be defined as:

(2) $$f^{*}\left(u\right)=u\;f\;\left(\frac{1}{u}\right)\;\;\;\mathrm{for}\;\;\;u\in R_{0}.$$

If f∈F is strictly convex at 1 with f(1)=0, and

$$\exists c\in R\;|\;f^{*}\left(u\right)=f\left(u\right)+c\left(u-1\right),$$

then $D_{f}\left(P,Q\right)$ satisfies the following basic properties [28,29,30]:

1. *Non-negativity and identity of indiscernibles*: $D_{f}\left(P,Q\right)\geq 0$ with $D_{f}\left(P,Q\right)=0$⇔P=Q,
2. *Symmetry*: $D_{f}\left(P,Q\right)=D_{f}\left(Q,P\right)$,
3. *Range of values*: $f\left(1\right)\leq D_{f}\left(P,Q\right)\leq f\left(0\right)+\lim_{t\to 0^{+}}f^{*}\left(t\right)$.

Given a set of functions f(u), the corresponding Csiszár divergences may not be different. In this regard, we have the following property.

4. *Uniqueness*: $D_{f_{1}}\left(P,Q\right)=D_{f}\left(P,Q\right)$, $\Leftrightarrow \exists c\in R\;|\;f_{1}\left(u\right)=f\left(u\right)+c\left(u-1\right)$.

Now, let us consider how to obtain true distances from symmetric Csiszár divergences. Refs. [29,30,31] proved and analyzed a necessary condition for a symmetric Csiszár divergence to satisfy the triangle inequality.

**Theorem 1** (Csiszár True Distances)**.** Let $D_{f}\left(P,Q\right)$ be a Csiszár divergence, such that f(u) is strictly convex at *1*, f(1)=0, and $f^{*}\left(u\right)=f\left(u\right)$ (required for symmetry under P↔Q).

*If the function*

(3) $$h_{\alpha }\left(u\right)=\frac{{\left(1-u^{\alpha }\right)}^{1/\alpha }}{f(u)}$$

*is nonincreasing on u∈[0,1) for $\alpha \in R_{0}$, then*

(4) $$\begin{array}{c} d_{\alpha }\left(P,Q\right)≐{\left[D_{f}\left(P,Q\right)\right]}^{\alpha } \end{array}$$

*satisfies the triangle inequality, i.e., $d_{\alpha }\left(P,Q\right)\leq d_{\alpha }\left(P,R\right)+d_{\alpha }\left(R,Q\right)$. Correspondingly, $d_{\alpha }\left(P,Q\right)$ is a true distance in the space of probability distributions [29,30,31].*

In the next section, we introduce quantum Csiszár distances based on the previously introduced Csiszár divergences for those f(u) leading to symmetric quantities, and we show how to obtain true distances in the space of quantum states by using Theorem 1.

## 3. Quantum Csiszár Distances

Let us address the problem of deriving quantum distances starting from symmetric Csiszár divergences between probability distributions. In the first place, we introduce the *quantum Csiszár distance* between pure quantum states and prove that it belongs to the class of distinguishability measures in the quantum realm, i.e., distance measures based on a measurement procedure [11]. Based on the previous result, we introduce a new family of distinguishability quantifiers for arbitrary (even mixed) quantum states, by taking the minimum quantum Csiszár distance over all possible purifications of the states to be distinguished, leading in this way to a well-defined quantum distance.

Let us consider then the following definition.

**Definition 1** (Quantum Csiszár Distance)**.** *Let f∈F be a differentiable strictly convex function at *1*, satisfying,*

*a*. *f(1)=0,*
*b*. ∃c∈R, such that $f^{*}\left(u\right)=f\left(u\right)+c\left(u-1\right)$ for all u∈[0,∞),
*c*. *$uf^{\prime }\left(u\right)\leq c$ for all u∈[0,1].*

*Given two pure states $|\psi_{p}\rangle$ and $|\psi_{q}\rangle$, the quantum Csiszár distance is*

(5) $$\begin{array}{c} D_{f}\left(|\psi_{p}\rangle ,|\psi_{q}\rangle \right)≐\left(1-\sqrt{1-{\left|\langle \psi_{p}|\psi_{q}\rangle \right|}^{2}}\right)f\left(\frac{1+\sqrt{1-{\left|\langle \psi_{p}|\psi_{q}\rangle \right|}^{2}}}{1-\sqrt{1-{\left|\langle \psi_{p}|\psi_{q}\rangle \right|}^{2}}}\right). \end{array}$$

Before addressing and defining the extension of this quantity to arbitrary mixed states, let us see how the previous quantity arises from an optimization procedure over a set of measurements related to the states to be distinguished, having a clear interpretation as a distinguishability measure based on measurements.

Let us consider two arbitrary quantum states represented by $|\psi_{p}\rangle$ and $|\psi_{q}\rangle$ belonging to a finite-dimensional Hilbert space. Following the general ideas introduced in Ref. [12], a natural starting point to define distinguishability measures can be summarized in the next three steps:

1. Take an arbitrary symmetric Csiszár divergence $D_{f}$ (cf. Equation (1)) between two classical probability distributions (see Section 2.1.1 for the properties of the defining Csiszár function f(u)).
2. Evaluate $D_{f}$ between the two classical probability distributions resulting from taking a measurement over the quantum system in either state $|\psi_{p}\rangle$ or $|\psi_{q}\rangle$.
3. Carry out an optimization of the resulting quantity obtained in the previous step over a set of measurements performed upon the quantum system.

In general terms, if we consider an arbitrary measurement $E={\left\{E_{i}\right\}}_{i=1}^{K}$ given by the *Positive Operator-Valued Measurement* (POVM) formalism [14,32], for arbitrary quantum states ρ and σ, the probability distributions for the outcomes of E are:

(6) $$\begin{array}{ccc} P_{E} & = & {\left\{p_{i}|p_{i}=\;\mathrm{Tr}\left(E_{i}\rho \right)\right\}}_{i}, \end{array}$$

(7) $$\begin{array}{ccc} Q_{E} & = & {\left\{q_{i}|q_{i}=\;\mathrm{Tr}\left(E_{i}\sigma \right)\right\}}_{i}, \end{array}$$

respectively.

Given the complexity involved when one tries to solve the corresponding optimization of Step 3 in the general case of POVMs, we shall focus on the optimization problem in the case of pure states and for the subset of POVMs defined by projective measurements (PVMs) represented by rank-1 projectors (also known as von Neumann measurements [32]) in the subspace defined by the states to be distinguished. In this regard, we state the following result.

**Theorem** **2.** *Let $|\psi_{p}\rangle$ and $|\psi_{q}\rangle$ be two pure quantum states. Let f(u) be a Csiszár function, such that $D_{f}$ stands for a quantum Csiszár distance, see Definition 1. Then,*

(8) $$\max_{E_{0}}D_{f}\left(P_{E_{0}},Q_{E_{0}}\right)=\left(1-\sqrt{1-{\left|\langle \psi_{p}|\psi_{q}\rangle \right|}^{2}}\right)f\left(\frac{1+\sqrt{1-{\left|\langle \psi_{p}|\psi_{q}\rangle \right|}^{2}}}{1-\sqrt{1-{\left|\langle \psi_{p}|\psi_{q}\rangle \right|}^{2}}}\right),$$

*with $P_{E_{0}}={\left\{p_{i}|p_{i}=\;Tr(E_{i}|\psi_{p}\rangle \langle \psi_{p}|)\right\}}_{i}$, $Q_{E_{0}}={\left\{q_{i}|q_{i}=\;Tr(E_{i}|\psi_{q}\rangle \langle \psi_{q}|)\right\}}_{i}$, and $E_{0}={\left\{E_{i}\right\}}_{i=1}^{2}$ is a projective measurement onto the subspace spanned by $|\psi_{p}\rangle$ and $|\psi_{q}\rangle$.*

**Proof.**  Given arbitrary $|\psi_{p}\rangle$ and $|\psi_{q}\rangle$ satisfying

$$|\langle \psi_{p}|\psi_{q}\rangle |=\cos\phi ,$$

we have to optimize $D_{f}\left(P_{E_{0}},Q_{E_{0}}\right)$ over the projective measurements $E_{0}={\left\{E_{i}\right\}}_{i=1}^{2}$ onto the subspace defined by the states to be distinguished. The connection between all these elements is summarized by the following parametrization [33,34,35,36],

(9) $$\begin{array}{cc} |\psi_{p}\rangle & =\cos\theta_{p}|e_{1}\rangle +\sin\theta_{p}|e_{2}\rangle , \end{array}$$

(10) $$\begin{array}{cc} |\psi_{q}\rangle & =\sin\theta_{q}|e_{1}\rangle +\cos\theta_{q}|e_{2}\rangle , \end{array}$$

with $E_{i}=|e_{i}\rangle \langle e_{i}|$, i∈{1,2}, and $|e_{1}\rangle$ and $|e_{2}\rangle$ are two orthogonal states. As $|\langle \psi_{p}|\psi_{q}\rangle |=\cos\left(\frac{\pi }{2}-\theta_{p}-\theta_{q}\right)$ holds, we have to impose the following constraints for $\theta_{p}$ and $\theta_{q}$,

(11) $$\begin{array}{cc} \; & 0\leq \theta_{p}\leq \pi /2, \end{array}$$

(12) $$\begin{array}{cc} \; & 0\leq \theta_{q}\leq \pi /2, \end{array}$$

(13) $$\begin{array}{cc} \; & \theta_{p}+\theta_{q}+\phi =\pi /2. \end{array}$$

At this point, and because of the symmetries of the optimization problem, it is convenient to take a fixed measurement and to optimize over the angles $\theta_{p}$ and $\theta_{q}$ subject to the constraints (11)–(13). Thus, the probabilities associated with each possible outcome (i.e., after the von Neumann measurement $\{|e_{i}\rangle {\langle e_{i}|\}}_{i}$ is performed on the quantum system) are given by the following equations:

(14) $$\begin{array}{cc} \; & p_{1}≐p≐\;\mathrm{Tr}(E_{1}|\psi_{p}\rangle \langle \psi_{p}|)=\cos^{2}\theta_{p}, \end{array}$$

(15) $$\begin{array}{cc} \; & p_{2}=1-p, \end{array}$$

(16) $$\begin{array}{cc} \; & q_{1}≐q≐\;\mathrm{Tr}(E_{1}|\psi_{q}\rangle \langle \psi_{q}|)=\sin^{2}\theta_{q}, \end{array}$$

(17) $$\begin{array}{cc} \; & q_{2}=1-q. \end{array}$$

The optimization problem over the PVMs turns out to be equivalent to finding the values of $\theta_{p}$ and $\theta_{q}$ maximizing the following equation:

(18) $$\begin{array}{c} D_{f}\left(P_{E_{0}},Q_{E_{0}}\right)=\sin^{2}\theta_{q}\;f\;\left(\frac{\cos^{2}\theta_{p}}{\sin^{2}\theta_{q}}\right)+\cos^{2}\theta_{q}\;f\;\left(\frac{\sin^{2}\theta_{p}}{\cos^{2}\theta_{q}}\right), \end{array}$$

subject to the constraints (11)–(13). Let us apply the method of Lagrange multipliers to this particular parametrization. The resulting Lagrangian can be written in the following way:

(19) $$L\left(\theta_{p},\theta_{q},\lambda \right)=D_{f}\left(\theta_{p},\theta_{q}\right)-\lambda \;\left(\theta_{p}+\theta_{q}+\phi -\pi /2\right).$$

The critical points of $L(\theta_{p},\theta_{q},\lambda )$ need to satisfy the following set of equations:

(20) $$\begin{array}{c} \frac{\partial L}{\partial \theta_{p}}=\frac{\partial L}{\partial \theta_{q}}=\frac{\partial L}{\partial \lambda }=0. \end{array}$$

After some calculations, we arrive at

(21) $$\begin{array}{cc} \; & \left[f^{\prime }\left(\frac{\sin^{2}\left(\theta_{p}\right)}{\cos^{2}\left(\theta_{q}\right)}\right)-f^{\prime }\left(\frac{\cos^{2}\left(\theta_{p}\right)}{\sin^{2}\left(\theta_{q}\right)}\right)\right]\;\sin\left(2\theta_{p}\right)=\\ \; & \left[f^{\prime }\left(\frac{\sin^{2}\left(\theta_{q}\right)}{\cos^{2}\left(\theta_{p}\right)}\right)-f^{\prime }\left(\frac{\cos^{2}\left(\theta_{q}\right)}{\sin^{2}\left(\theta_{p}\right)}\right)\right]\;\sin\left(2\theta_{q}\right). \end{array}$$

Using the properties of f(u) and the mirror symmetry around $\theta_{0}=\frac{\pi }{4}-\frac{\phi }{2}$, it can be seen that Equation (21) admits a unique solution of the form $\theta_{p}^{\mathrm{opt}}=\theta_{q}^{\mathrm{opt}}=\theta_{0}$.

It remains to prove that the previous critical point corresponds to a maximum value of $D_{f}\left(P_{E_{0}},Q_{E_{0}}\right)$. To achieve this, let us introduce $\theta_{q}=\pi /2-\phi -\theta_{p}$ in Equation (18), obtaining:

$$\begin{array}{c} D_{f}\left(P_{E_{0}},Q_{E_{0}}\right)=\cos^{2}\theta_{p}\;f\;\left[\frac{\cos^{2}\left(\phi +\theta_{p}\right)}{\cos^{2}\theta_{p}}\right]+\sin^{2}\theta_{p}\;f\;\left[\frac{\sin^{2}\left(\phi +\theta_{p}\right)}{\sin^{2}\theta_{p}}\right]=:g\left(\theta_{p}\right), \end{array}$$

which, additionally, is invariant under the transformation $\theta_{p}\to 2\theta_{0}-\theta_{p}$. Therefore, by considering that we have only one critical point, $\theta_{p}=\theta_{0}$, the maximum condition is demonstrated, if we prove

$$\frac{\partial g(\theta_{p})}{\partial \theta_{p}}{|}_{\theta_{p}=0}\geq 0.$$

Carrying out the required calculations, we can show that the previous inequality holds for an arbitrary function f(u) satisfying $uf^{\prime }\left(u\right)\leq c$ for all u∈[0,1]. Finally, the resulting maximum is:

(22) $$\max_{E_{0}}D_{f}\left(P_{E_{0}},Q_{E_{0}}\right)=\left(1-\sin\phi \right)f\;\left(\frac{1+\sin\phi }{1-\sin\phi }\right).$$

We arrive to the final result by noting that $\cos\phi =\left|\langle \psi_{p}|\psi_{q}\rangle \right|$; therefore, $\sin\phi =\sqrt{1-{\left|\langle \psi_{p}|\psi_{q}\rangle \right|}^{2}}$. □

Having introduced the quantum Csiszár distance and established its operational interpretation as a one-shot distinguishability measure [11], let us propose one interesting extension of $D_{f}$ to the general case of mixed states.

**Definition 2** (Extended Quantum Csiszár Distance)**.** *Let us define*

(23) $$\begin{array}{c} \Phi_{f}\left(x\right)≐\left(1-\sqrt{1-x}\right)f\left(\frac{1+\sqrt{1-x}}{1-\sqrt{1-x}}\right), \end{array}$$

*with f(u), such that $D_{f}$ is a quantum Csiszár distance, see Definition 1.*

*The extended quantum Csiszár distance between two arbitrary states ρ and σ, defined over a finite-dimensional Hilbert space, is*

(24) $$\begin{array}{c} D_{f}^{\mathrm{ext}}\left(\rho ,\sigma \right)≐\Phi_{f}\left[F(\rho ,\sigma )\right], \end{array}$$

*with*

(25) $$\begin{array}{c} F\left(\rho ,\sigma \right)≐{\left[\;Tr\left(\sqrt{\sqrt{\rho }\sigma \sqrt{\rho }}\right)\right]}^{2}, \end{array}$$

*as the Uhlmann–Jozsa fidelity.*

The previous quantity $D_{f}^{\mathrm{ext}}$ has a clear and interesting interpretation. It is the minimal quantum Csiszár distance over all possible *purifications* of the quantum states ρ and σ to be distinguished. We can see this clearly by considering

(26) $$\begin{array}{c} F\left(\rho ,\sigma \right)=\max_{|\psi \rangle ,|\phi \rangle }{\left|\langle \psi |\phi \rangle \right|}^{2}, \end{array}$$

where |ψ〉 and |ϕ〉 are purifications of ρ and σ, respectively. Taking into account that $\Phi_{f}\left(x\right)$ is a monotonic-decreasing function, it follows that:

(27) $$\begin{array}{c} D_{f}^{\mathrm{ext}}\left(\rho ,\sigma \right)=\min_{|\psi \rangle ,|\phi \rangle }D_{f}(|\psi \rangle ,|\phi \rangle )=\min_{|\psi \rangle ,|\phi \rangle }\Phi_{f}\left({\left|\langle \psi |\phi \rangle \right|}^{2}\right)=\Phi_{f}\left[F(\rho ,\sigma )\right]. \end{array}$$

### 3.1. Quantum Csiszár True Distances

Now, let us explore how we can derive quantum Csiszár true distances using the quantum Csiszár distances $D_{f}$, as defined in Equation (5). For any two arbitrary quantum states ρ and σ, we have demonstrated that the quantity $D_{f}^{\mathrm{ext}}\left(\rho ,\sigma \right)$ is linked, through the purification of quantum states, to an optimization process over projective measurements. This ultimately leads to a correspondence between the quantum states to be distinguished and the probability distribution for the outcomes of the optimal measurement, i.e., in general terms, $\rho ↔P_{E_{\mathrm{opt}}}$ and $\sigma ↔Q_{E_{\mathrm{opt}}}$. This correspondence enables us to construct quantum Csiszár distances that satisfy the triangle inequality by means of Theorem 1. The following proposition summarizes the preceding concepts:

**Proposition** **1.** *Let f(u) be a function defining a quantum Csiszár distance (cf. Definition 1). If, additionally,*

(28) $$\begin{array}{c} \left(u-u^{1-\alpha }\right)f^{\prime }\left(u\right)\leq f\left(u\right), \end{array}$$

*for all u∈(0,1) and a fixed real positive number α, then*

(29) $$\begin{array}{c} d_{f,\alpha }\left(\rho ,\sigma \right)={\left[D_{f}^{\mathrm{ext}}\left(\rho ,\sigma \right)\right]}^{\alpha } \end{array}$$

*satisfies the triangle inequality and, therefore, is a true distance in the space of pure quantum states.*

**Proof.**  We have to prove the triangle inequality. Given the general correspondence between quantum states and probability distributions $\rho ↔P_{E_{\mathrm{opt}}}$ and $\sigma ↔Q_{E_{\mathrm{opt}}}$, implied by Theorem 2 and the purification procedure, we just have to apply Theorem 1. Therefore, we have to find α>0 such that $h_{\alpha }\left(u\right)$ is a non-increasing function on the interval [0,1). In our case, this is equivalent to $h_{\alpha }^{\prime }\left(u\right)\leq 0$ for u∈(0,1) (which immediately leads to $h_{\alpha }^{\prime }\left(0\right)\leq 0$). The calculation is

(30) $$\begin{array}{c} \frac{\partial h_{\alpha }\left(u\right)}{\partial u}=\frac{{\left(1-u^{\alpha }\right)}^{\frac{1-\alpha }{\alpha }}\left[\left(u^{\alpha +1}-u\right)f^{\prime }\left(u\right)-u^{\alpha }f\left(u\right)\right]}{uf{\left(u\right)}^{2}}. \end{array}$$

The sign of $h_{\alpha }^{\prime }$ then is ruled by $\left(u^{\alpha +1}-u\right)f^{\prime }\left(u\right)-u^{\alpha }f\left(u\right)$. Therefore, $h_{\alpha }\left(u\right)$ is non-increasing, if and only if $\left(u-u^{1-\alpha }\right)f^{\prime }\left(u\right)\leq f\left(u\right)$ for all u∈(0,1). □

As final remarks, we have seen that Proposition 1 allows one to obtain a family of distances satisfying the triangle inequality between quantum states defined over a Hilbert space of arbitrary (but finite) dimension. Mainly because of its generality, this is a potentially powerful tool for finding new informational inequalities.

Regarding the properties of the resulting quantum Csiszár true distances $d_{f,\alpha }\left(\rho ,\sigma \right)$ (cf. Equation (29)), as they are functions of the Uhlmann–Jozsa fidelity, they inherit all the properties of F(ρ,σ), for example, the monotonicity under completely positive trace-preserving quantum operations.

### 3.2. Examples

In this section, as examples of application, we apply the main result of this work to some specific *f*-divergences between two probability distributions $P={\left\{p_{i}\right\}}_{i=1}^{n}$ and $Q={\left\{q_{j}\right\}}_{j=1}^{n}$. In addition, in Table 1, we have included the expressions of the resulting quantum Csiszár true distances with their corresponding range of suitable α values, given by Proposition 1 and Equation (28).

#### 3.2.1. Variational Distance

The *variational distance* (often referred to as the *trace distance* or *Kolmogorov distance* [32]) between two probability distributions *P* and *Q* is defined as,

(31) $$D_{V}\left(P,Q\right)=\frac{1}{2}\sum_{i=1}^{n}\left|p_{i}-q_{i}\right|.$$

This distance belongs to the class of *f*-divergences (cf. Equation (1)). The corresponding function $f_{V}\left(u\right)$ is given by:

(32) $$f_{V}\left(u\right)=\frac{1}{2}\left|u-1\right|.$$

It is straightforward to verify that $f_{V}^{*}\left(u\right)-f_{V}\left(u\right)=0$. Thus, the variational distance fulfills the basic Csiszár properties 1–3 in Section 2.1.1, for c=0, leading to a well-defined *distance* (see Section 2) between probability distributions. Moreover, it is worth mentioning that the trace distance also defines a true distance, as it satisfies all the Properties 1–4 (cf. Section 2).

The corresponding quantum Csiszár distance $D_{V}$ between two pure quantum states $|\psi_{p}\rangle$ and $|\psi_{q}\rangle$, obtained via the optimization problem summarized in Theorem 2, is

(33) $$\begin{array}{c} D_{V}\left(|\psi_{p}\rangle ,|\psi_{q}\rangle \right)≐\sqrt{1-{\left|\langle \psi_{p}|\psi_{q}\rangle \right|}^{2}}. \end{array}$$

Given that $D_{V}\left(P,Q\right)$ satisfies the triangle inequality, $D_{V}$ is also a true distance between quantum states.

On the other hand, the so-called quantum trace distance between two density operators ρ and σ, already introduced in the literature, is an example of a distance induced by a norm, i.e., the trace norm of an operator or Schatten $L_{1}$-norm. Indeed, the trace distance is defined as

(34) $$D_{tr}\left(\rho ,\sigma \right)≐\frac{1}{2}||\rho -{\sigma ||}_{1}≐\frac{1}{2}\;\mathrm{Tr}\left|\rho -\sigma \right|,$$

where $\left|A\right|=\sqrt{A^{†}A}$. Notably, this quantity can also be obtained from its classical form as a distance measure between two probability distributions (see Equation (31)) by means of the optimization over arbitrary POVM measurements described at the beginning of Section 3. Particularly, this distance constitutes an example of a procedure to define a quantum distinguishability measure when a single measurement process is involved. In this case, the problem at hand is to solve:

(35) $$\max_{E}D_{V}\left(P_{E},Q_{E}\right),$$

where the maximum is taken over all possible POVMs. The result of this maximization procedure is summarized in the following theorem due to C. W. Helstrom [14,37]:

**Theorem 3**  ((Helstrom’s) [37])**.*** Let $p_{i}=\;Tr\left(E_{i}\rho \right)$, and $q_{i}=\;Tr\left(E_{i}\sigma \right)$. Then,*

(36) $$\max_{E}D_{V}\left(P_{E},Q_{E}\right)=\frac{1}{2}\;Tr\left|\rho -\sigma \right|=D_{tr}\left(\rho ,\sigma \right),$$

*where the maximization is carried out over all POVMs.*

Remarkably, when we consider the pure quantum states $\rho =|\psi_{p}\rangle \langle \psi_{p}|$ and $\sigma =|\psi_{q}\rangle \langle \psi_{q}|$, the quantum trace distance turns out to be equal to the quantum Csiszár distance corresponding to the variational distance, i.e.,

$$\begin{array}{c} D_{tr}\left(|\psi_{p}\rangle ,|\psi_{q}\rangle \right)=\sqrt{1-{\left|\langle \psi_{p}|\psi_{q}\rangle \right|}^{2}}=D_{V}\left(|\psi_{p}\rangle ,|\psi_{q}\rangle \right). \end{array}$$

As we can see, in this case, the maximization over arbitrary POVMs leads to the same result as the optimization over the set of projective measurements we have employed in Theorem 2.

It remains to analyze the differentiability of $f_{V}$. Clearly, this function is not derivable at u=1. However, this fact does not affect our result in Theorem 2 because of the constraint in Equation (13).

The extended quantum Csiszár distance resulting from the variational distance is

(37) $$\begin{array}{c} D_{V}^{\mathrm{ext}}\left(\rho ,\sigma \right)=\sqrt{1-F(\rho ,\sigma )}. \end{array}$$

On the other hand, by introducing $f_{V}$ in Equation (28), it is possible to show that

(38) $$\begin{array}{c} d_{V,\alpha }\left(\rho ,\sigma \right)={\left[1-F(\rho ,\sigma )\right]}^{\frac{\alpha }{2}} \end{array}$$

turns out to be a true distance in the space of quantum states for α∈(0,1].

#### 3.2.2. Hellinger Discrimination

The *Hellinger discrimination* between the two probability distributions *P* and *Q* is defined as [38,39]:

(39) $$D_{H}\left(P,Q\right)=\frac{1}{2}\sum_{i=1}^{n}{\left|\sqrt{p_{i}}-\sqrt{q_{i}}\right|}^{2}.$$

The corresponding function f(u) is given by

(40) $$f_{H}\left(u\right)=\frac{1}{2}{\left(\sqrt{u}-1\right)}^{2}.$$

As $f_{H}^{*}\left(u\right)-f_{H}\left(u\right)=0$ holds, the conditions of Theorem 2 are satisfied for c=0. The quantum Csiszár distance (cf. Definition 1) given by $f_{H}\left(u\right)$ is

(41) $$\begin{array}{c} D_{H}\left(|\psi_{p}\rangle ,|\psi_{q}\rangle \right)≐1-\left|\langle \psi_{p}|\psi_{q}\rangle \right|. \end{array}$$

The previous quantity is not the only existing quantum extension of the Hellinger discrimination. Ref. [38] introduced, by natural extension of the classical case, the *Hellinger distance,*

(42) $$\begin{array}{c} H_{e}\left(\rho ,\sigma \right)≐1-A\left(\rho ,\sigma \right)\;\;\mathrm{with}\;\;A\left(\rho ,\sigma \right)≐\;\mathrm{Tr}\sqrt{\rho }\sqrt{\sigma }, \end{array}$$

as an informational distance on the quantum states’ space. The quantity A(ρ,σ) is called quantum affinity, and it stands for the quantum extension of $A\left(P,Q\right)=\sum_{i}\sqrt{p_{i}q_{i}}$. Note that we have discarded the factor 2 in the expressions of $H_{e}$ and *A* (see Ref. [38]). The reason is that we have the factor 1/2 in the definition of the classical distance (cf. Equation (39)). In this way, if the quantum states ρ and σ do commute, then $H_{e}$ does coincide with $D_{H}$.

In the case of pure quantum states, $A\left(|\psi_{p}\rangle ,|\psi_{q}\rangle \right)={\left|\langle \psi_{p}|\psi_{q}\rangle \right|}^{2}$; therefore, $H_{e}\left(|\psi_{p}\rangle ,|\psi_{q}\rangle \right)=1-{\left|\langle \psi_{p}|\psi_{q}\rangle \right|}^{2}$. As we can see, $H_{e}\left(|\psi_{p}\rangle ,|\psi_{q}\rangle \right)>D_{H}\left(|\psi_{p}\rangle ,|\psi_{q}\rangle \right)$ for non-orthogonal quantum states.

For the Hellinger discrimination, the extended quantum Csiszár distance is

(43) $$\begin{array}{c} D_{H}^{\mathrm{ext}}\left(\rho ,\sigma \right)=1-\sqrt{F(\rho ,\sigma )}. \end{array}$$

In addition, taking into account Equation (28) in Proposition 1, we have that for α∈(0,1/2],

(44) $$\begin{array}{c} d_{H,\alpha }\left(\rho ,\sigma \right)={\left[1-\sqrt{F(\rho ,\sigma )}\right]}^{\alpha } \end{array}$$

is a true distance in the space of quantum states.

#### 3.2.3. Triangular Discrimination

The *triangular discrimination* between the two probability distributions *P* and *Q* is defined as [40,41]

(45) $$D_{T}\left(P,Q\right)=\frac{1}{2}\sum_{i=1}^{n}\frac{|p_{i}-q_{i}{|}^{2}}{p_{i}+q_{i}},$$

and it is a symmetrized version of the chi-square divergence [42], also known as the *Le Cam divergence [43]. The corresponding function f(u) defining this Csiszár divergence can be written as a Csiszár divergence by taking the corresponding function f(u) as*

(46) $$f_{T}\left(u\right)=\frac{1}{2}\frac{{\left(u-1\right)}^{2}}{u+1}.$$

As in the previous cases, it is straightforward to see that $f_{T}^{*}\left(u\right)=f_{T}\left(u\right)$; therefore, the triangular discrimination satisfies the conditions of Theorem 2 for c=0.

The Csiszár quantum distance associated with this notion of distinguishability is (cf. Definition 1)

(47) $$\begin{array}{c} D_{T}\left(|\psi_{p}\rangle ,|\psi_{q}\rangle \right)≐1-{\left|\langle \psi_{p}|\psi_{q}\rangle \right|}^{2}. \end{array}$$

To the best of our knowledge, there is no other quantum extension based on the triangular discrimination $D_{T}$. Nevertheless, it is noteworthy that

(48) $$\begin{array}{c} D_{T}\left(|\psi_{p}\rangle ,|\psi_{q}\rangle \right)=H_{e}\left(|\psi_{p}\rangle ,|\psi_{q}\rangle \right) \end{array}$$

holds, where $H_{e}\left(|\psi_{p}\rangle ,|\psi_{q}\rangle \right)$ is the Hellinger distance proposed in Ref. [38] by Luo and Zhang.

The extended quantum Csiszár distance for the triangular discrimination can be expressed as follows:

(49) $$\begin{array}{c} D_{T}^{\mathrm{ext}}\left(\rho ,\sigma \right)=1-F\left(\rho ,\sigma \right). \end{array}$$

In addition, Equation (28) implies that for any α∈(0,1/2], the expression,

(50) $$\begin{array}{c} d_{T,\alpha }\left(\rho ,\sigma \right)={\left[1-F(\rho ,\sigma )\right]}^{\alpha }, \end{array}$$

fulfills the triangle inequality in the quantum-state space. As we can see, the quantum Csiszár true distances given by the Hellinger and the variational distance are equivalent, although the corresponding quantum Csiszár distances are different.

#### 3.2.4. Jensen–Shannon Divergence

The *Jensen–Shannon divergence* between two probability distributions *P* and *Q* can be written in the following form [44]:

(51) $$D_{\mathrm{JS}}\left(P,Q\right)=\frac{1}{2}\left[D_{\mathrm{KL}}\left(P,\frac{P+Q}{2}\right)+D_{\mathrm{KL}}\left(Q,\frac{P+Q}{2}\right)\right],$$

where $D_{\mathrm{KL}}\left(P,Q\right)=\sum_{i}p_{i}\log_{2}\frac{pi}{qi}$ is the Kullback–Leibler divergence. The Csiszár function corresponding to the Jensen–Shannon divergence is

(52) $$f_{\mathrm{JS}}\left(u\right)=\frac{1}{2}\left[\left(1+u\right)+u\log_{2}\left(u\right)-\left(1+u\right)\log_{2}\left(1+u\right)\right]$$

for $u\in R_{+}$. Additionally, this Csiszár divergence satisfies $f_{\mathrm{JS}}^{*}\left(u\right)=f_{\mathrm{JS}}\left(u\right)$; therefore, the basic properties for having a distance in the space of probability distributions are fulfilled for c=0 (see Section 2.1.1). Moreover, in the classical case, it was shown that the square root of $D_{\mathrm{JS}}\left(P,Q\right)$ satisfies the triangle inequality [45,46,47,48].

The resulting quantum Csiszár distance corresponding to the Jensen–Shannon divergence can be written in terms of the binary entropy function $H\left(p\right)≐-p\log_{2}\left(p\right)-\left(1-p\right)\log_{2}\left(1-p\right)$,

(53) $$\begin{array}{c} D_{\mathrm{JS}}\left(|\psi_{p}\rangle ,|\psi_{q}\rangle \right)=1-H\left(\frac{1-\sqrt{1-{\left|\langle \psi_{p}|\psi_{q}\rangle \right|}^{2}}}{2}\right). \end{array}$$

This particular measure of distinguishability was analyzed in Ref. [12] in which it was shown that $D_{\mathrm{JS}}\left(|\psi_{p}\rangle ,|\psi_{q}\rangle \right)$ is equal to the *accessible information* associated with the ensemble $\left\{|\psi_{p}\rangle ,|\psi_{q}\rangle \right\}$, for the equiprobable case, and given by the projection measurement we considered in Theorem 2.

Another quantum extension of the Jensen–Shannon divergence was proposed in Ref. [44] by natural extension of the classical quantity. The *quantum Jensen–Shannon divergence* (QJSD) can be written as:

(54) $$\begin{array}{c} \mathrm{QJSD}\left(\rho ,\sigma \right)≐\frac{1}{2}\left[S_{r}\left(\rho ||\bar{\rho }\right)+S_{r}\left(\rho ||\bar{\rho }\right)\right], \end{array}$$

where $\bar{\rho }=\frac{\rho +\sigma }{2}$, and $S_{r}\left(\rho ||\sigma \right)=\;\mathrm{Tr}\rho \left(\log_{2}\rho -\log_{2}\sigma \right)$ is the quantum relative entropy. For pure quantum states, the QJSD reduces to

(55) $$\begin{array}{c} \mathrm{QJSD}\left(|\psi_{p}\rangle ,|\psi_{q}\rangle \right)=H\left(\frac{1-\left|\langle \psi_{p}|\psi_{q}\rangle \right|}{2}\right). \end{array}$$

Additionally, the QJSD is the Holevo bound (often called Holevo information) related to the ensemble {ρ,σ}, in the equiprobable case.

The extended quantum Csiszár distance for the Jensen–Shannon divergence is given by:

(56) $$\begin{array}{c} D_{\mathrm{JS}}^{\mathrm{ext}}\left(\rho ,\sigma \right)=1-H\left(\frac{1-\sqrt{1-F(\rho ,\sigma )}}{2}\right). \end{array}$$

Finally, bearing in mind Proposition 1, it is easy to show that

(57) $$\begin{array}{c} d_{\mathrm{JS},\alpha }\left(\rho ,\sigma \right)={\left[1-H\left(\frac{1-\sqrt{1-F(\rho ,\sigma )}}{2}\right)\right]}^{\alpha } \end{array}$$

defines a true distance in the space of quantum states for α∈(0,1/2]. This was firstly addressed in Ref. [12].

#### 3.2.5. Comparison between $D_{f}^{\max}$ and $D_{f}^{\mathrm{ext}}$

Given two arbitrary quantum states ρ and σ, let us define

(58) $$\begin{array}{c} D_{f}^{\max}\left(\rho ,\sigma \right)≐\max_{E_{1}}D_{f}\left(P_{E_{1}},Q_{E_{1}}\right) \end{array}$$

where $E_{1}$ is a von Neumann measurement, and $P_{E_{1}}$ and $Q_{E_{1}}$ are the probability distributions given by Equations (6) and (7), respectively.

**Figure 1:**
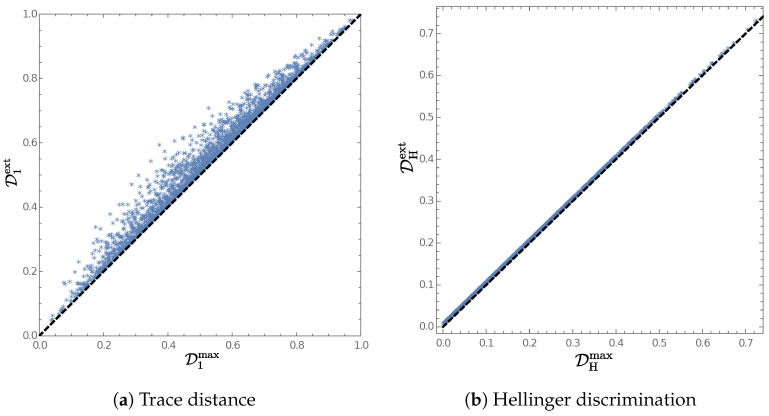

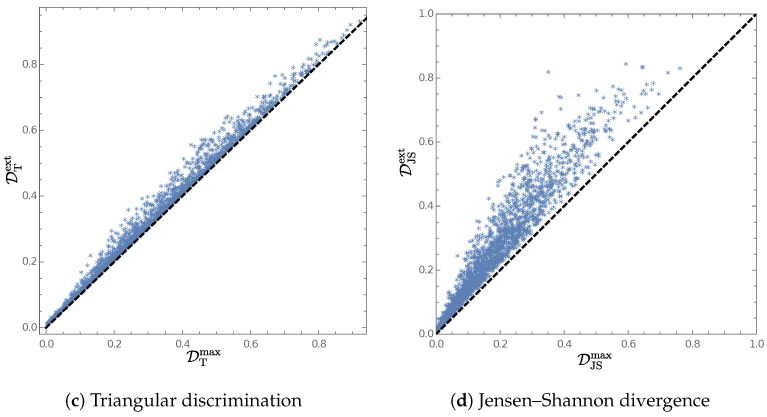
Comparison between $D_{f}^{\max}$ (cf. Equation (58)) and $D_{f}^{\mathrm{ext}}$ (cf. Equation (24)) for two thousand pairs of qubit quantum states, generated pseudo-randomly from the uniform distribution over the Bloch ball, for the symmetric Csiszár divergences considered in Section 3.2.

One may ask about the difference between $D_{f}^{\max}$ and $D_{f}^{\mathrm{ext}}$, defined in Equation (24). Based on the numerical simulation shown in Figure 1, we state the following conjecture:

**Conjecture** **1.** *Let ρ and σ be two quantum states defined over an n-dimensional Hilbert space.*

$$\begin{array}{c} D_{f}^{\mathrm{ext}}\left(\rho ,\sigma \right)\geq D_{f}^{\max}\left(\rho ,\sigma \right) \end{array}$$

*holds. In addition, in the case of the Hellinger discrimination (cf. Equation (39)), the equality*

$$\begin{array}{c} D_{H}^{\mathrm{ext}}\left(\rho ,\sigma \right)=D_{H}^{\max}\left(\rho ,\sigma \right) \end{array}$$

*holds.*

In Figure 1, we show the results of $D_{f}^{\max}$ and $D_{f}^{\mathrm{ext}}$ evaluated for two thousand pairs of qubit quantum states, generated pseudo-randomly according to the uniform distribution over the Bloch ball.

## 4. Concluding Remarks

In this paper, we extended previous results in order to develop a procedure to build quantum distances starting from a set of symmetric *f*-divergences used as measures of dissimilarity between two probability distributions. The procedure involved a set of measurements performed upon the system of interest. Starting from different initial quantum states, probability distributions were obtained in association with the different possible outcomes obtained after performing a measurement upon the quantum system. Given the complexity of the general problem, we focused on the problem of distinguishing between two given pure states in the case of measurements represented by the projective measurement of rank 1. As a consequence, the posed problem was expressed in the form of an optimization procedure for a given *f*-divergence between the probability distributions associated with the different possible outcomes, over the set of rank-1 projectors. Thus, the resulting quantum distance belongs to the set of distinguishability measures in the quantum realm [10,11]. We found a closed form for this optimization process (cf. Definition 1), which allows one to obtain quantum distances starting from a set of symmetric *f*-divergences (cf. Theorem 2). Next, we showed that these quantum distances could be extended to the case of mixed quantum states by means of a procedure known as *purification* (see Definition 2). As it has been shown that a purification process can be physically implemented, this last result implies that an operational interpretation could be given to the obtained distinguishability measures between quantum states. In addition, we also analyzed the possibility of defining quantum distances satisfying the triangle inequality (i.e., *true distances*) by revisiting a criterium to be fulfilled by a particular function f(u) defining a Csiszár divergence (cf. Section 2); this can be found in Proposition 1. Thus, in the case of symmetric *f*-divergences, we also obtained the quantum distances, which satified the triangle inequality. In order to present some specific examples, we applied the general result to some relevant cases of Csiszár divergences such as the *Variational Distance*, *Hellinger* and *triangular discrimination*, and the *Jensen–Shannon divergence* (see Section 3.2). In the case of the *Variational Distance* and the *Jensen–Shannon divergence*, we also verified that our procedure was in agreement with the known results. In addition, we demonstrated that the resulting quantum Csiszár true distances for the triangular discrimination and the variational distance were equivalent. In the case of the Hellinger discrimination, we compared the corresponding Csiszár quantum distance, Definition 1, with the Hellinger distance considered in Ref. [38], demonstrating that the one obtained in this work was lower than the one introduced in Ref. [38]. This was linked also to the case of triangular discrimination. The quantum Csiszár distance given by the triangular discrimination was equal to the Hellinger distance proposed in Ref. [38], showing a connection between the preceding quantity and the measurement procedure.

Additionally, we presented numerical simulations—involving pairs of qubit states pseudo-randomly distributed over the Bloch ball—contrasting two quantities: the extended quantum Csiszár distance, Equation (24), and the optimized Csiszár divergence over arbitrary von Neumann measurements, Equation (58). This calculation allowed us to propose a conjecture, i.e., extended quantum Csiszár distances are generally greater than or equal to the ones resulting from taking the maximum of the Csiszár divergence over arbitrary measurements (for example, the equality is attained in the case of the Hellinger discrimination), see Conjecture 1.

The derivation of true distances in the quantum realm induced by fidelity is interesting and clearly well motivated as these magnitudes find applications in a variety of research subjects related to quantum information theory and quantum information processing, such as non-locality, quantum correlations, open quantum systems, quantum phase transitions, quantum coherence, among others [23,49,50,51,52,53,54,55]. In particular, due to the fact that the quantum Csiszár true distances introduced in this work satisfy the triangle inequality and can be given an operational interpretation in terms of the measurement and purifications processes, they are promising quantities that could find applications in channel discrimination theory [16], the study of non-Markovianity and information backflow in open quantum systems [17,18], the evaluation of the presence of quantum correlations [19], and the definition of new distance measures in the space of quantum channels [20].

In addition, the well-known results of the harmonic analysis allow us to determine, at least in principle, a map from a metric space into a Hilbert space in such a way that the true distance between two points is equal to the distance between the corresponding points in the Hilbert space [46,56,57]. Therefore, we believe that our results may also contribute to strengthening the link between geometric aspects of information theory and quantum information.

## Figures and Tables

**Table 1:** Quantum Csiszár true distances $d_{f,\alpha }\left(\rho ,\alpha \right)$ and their corresponding suitable values of α, satisfying Proposition 1, Equation (28), for the exemplary symmetric Csiszár divergences treated in Section 3.2.

Csiszár Distance, f(u)	$d_{f,\alpha }\left(\rho ,\alpha \right)$	True Distance for
Variational distance $\frac{1}{2}\left|u-1\right|$	${\left[1-F(\rho ,\sigma )\right]}^{\frac{\alpha }{2}}$	α∈(0,1]
Hellinger discrimination $\frac{1}{2}{\left(\sqrt{u}-1\right)}^{2}$	${\left[1-\sqrt{F(\rho ,\sigma )}\right]}^{\alpha }$	α∈(0,1/2]
Triangular discrimination $\frac{1}{2}\frac{{\left(u-1\right)}^{2}}{u+1}$	${\left[1-F(\rho ,\sigma )\right]}^{\alpha }$	α∈(0,1/2]
Jensen–Shannon divergence $\frac{1}{2}[\left(1+u\right)+u\log_{2}\left(u\right)$ $-\left(1+u\right)\log_{2}\left(1+u\right)]$	${\left[1-H\left(\frac{1-\sqrt{1-F(\rho ,\sigma )}}{2}\right)\right]}^{\alpha }$	α∈(0,1/2]

## References

[1] Wilde M.M. (2017). Quantum Information Theory.

[2] Csiszár I. (1963). Eine informationstheoretische Ungleichung und ihre Anwendung auf den Beweis der Ergodizitat von Markoffschen Ketten. Magyar. Tud. Akad. Mat. Kutató Int. Közl.

[3] Ali S.M., Silvey S.D. (1966). A general class of coefficients of divergence of one distribution from another. J. R. Statist. Soc. Ser. B.

[4] Deza M., Deza E. (2016). Encyclopedia of Distances.

[5] Crooks G., Sivak D.A. (2011). Measures of trajectory ensemble disparity in nonequilibrium statistical dynamics. J. Stat. Mech..

[6] Hiai F., Mosonyi M., Petz D., Bény C. (2011). Quantum f-divergences and error correction. Rev. Math. Phys..

[7] Matsumoto K. (2018). A New Quantum Version of f-Divergence.

[8] Dragomir S.S. (2014). A new quantum f-divergence for trace class operators in hilbert spaces. Entropy.

[9] Hiai F., Petz D. (2012). From quasi-entropy to various quantum information quantities. Publ. Res. Inst. Math. Sci..

[10] Bussandri D.G., Lamberti P.W. (2020). Generalized Holevo theorem and distinguishability notions. J. Phys. A Math. Theor..

[11] Fuchs C.A. (1996). Distinguishability and Accessible Information in Quantum Theory. Ph.D. Thesis.

[12] Osán T.M., Bussandri D.G., Lamberti P.W. (2022). Quantum metrics based upon classical Jensen–Shannon divergence. Phys. A Stat. Mech. Its Appl..

[13] Galindo A., Martín-Delgado M.A. (2002). Information and computation: Classical and quantum aspects. Rev. Mod. Phys..

[14] Bengtsson I., Życzkowski K. (2006). Geometry of Quantum States: An Introduction to Quantum Entanglement.

[15] Bussandri D., Majtey A., Lamberti P.W., Osán T. (2019). Generalized approach to quantify correlations in bipartite quantum systems. Quantum Inf. Process..

[16] Bergh B., Datta N., Salzmann R., Wilde M.M. (2022). Parallelization of Sequential Quantum Channel Discrimination in the Non-Asymptotic Regime. arXiv.

[17] Megier N., Smirne A., Vacchini B. (2021). Entropic bounds on information backflow. Phys. Rev. Lett..

[18] Settimo F., Breuer H.P., Vacchini B. (2022). Entropic and trace-distance-based measures of non-Markovianity. Phys. Rev. A.

[19] Rudnicki Ł., Puchała Z., Horodecki P., Życzkowski K. (2014). Constructive entanglement test from triangle inequality. J. Phys. A Math. Theor..

[20] Gilchrist A., Langford N.K., Nielsen M.A. (2005). Distance measures to compare real and ideal quantum processes. Phys. Rev. A.

[21] Joshi P., Horodecki K., Horodecki M., Horodecki P., Horodecki R., Li B., Szarek S., Szarek T. (2015). Bound on Bell inequalities by fraction of determinism and reverse triangle inequality. Phys. Rev. A.

[22] Mendonça P.E., Napolitano R.d.J., Marchiolli M.A., Foster C.J., Liang Y.C. (2008). Alternative fidelity measure between quantum states. Phys. Rev. A.

[23] Ma Z., Zhang F.l., Chen J.l. (2009). Fidelity induced distance measures for quantum states. Phys. Lett. A.

[24] Hayashi M., Ishizaka S., Kawachi A., Kimura G., Ogawa T. (2015). Introduction to Quantum Information Science.

[25] Lin J. (1991). Divergence measures based on the Shannon entropy. IEEE Trans. Inform. Theory.

[26] Kullback S., Leibler R.A. (1951). On Information and Sufficiency. Ann. Math. Stat..

[27] Kullback S. (1968). Information Theory and Statistics.

[28] Vajda I. (1972). On *f*–divergence and singularity of probability measures. Period. Math. Hung..

[29] Kafka P., Oesterreicher F., Vincze I. (1991). On powers of *f*–divergences defining a distance. Stud. Sci. Math. Hung..

[30] Österreicher F., Vajda I. (2003). A new class of metric divergences on probability spaces and its applicability in statistics. Ann. Inst. Stat. Math..

[31] Österreicher F. (1996). On a class of perimeter-type distances of probability distributions. Kybernetika.

[32] Nielsen M.A., Chuang I.L. (2010). Quantum Computation and Quantum Information: 10th Anniversary Edition.

[33] Huttner B., Muller A., Gautier J.D., Zbinden H., Gisin N. (1996). Unambiguous quantum measurement of nonorthogonal states. Phys. Rev. A.

[34] Chefles A. (2000). Quantum state discrimination. Contemp. Phys..

[35] Rastegin A.E. (2002). Relative error of state-dependent cloning. Phys. Rev. A.

[36] Barnett S.M., Croke S. (2009). Quantum state discrimination. Adv. Opt. Photon..

[37] Helstrom C. (1976). Quantum Detection and Estimation Theory.

[38] Luo S., Zhang Q. (2004). Informational distance on quantum-state space. Phys. Rev. A.

[39] Dragomir S. (2002). Upper and lower bounds for Csiszár f-divergence in terms of Hellinger discrimination and applications. Nonlinear Anal. Forum.

[40] Topsoe F. (2000). Some inequalities for information divergence and related measures of discrimination. IEEE Trans. Inf. Theory.

[41] Foster D.J., Krishnamurthy A. (2021). Efficient first-order contextual bandits: Prediction, allocation, and triangular discrimination. Adv. Neural Inf. Process. Syst..

[42] Pearson K. (1900). On the criterion that a given system of deviations from the probable in the case of a correlated system of variables is such that it can be reasonably supposed to have arisen from random sampling. Lond. Edinb. Dublin Philos. Mag. J. Sci..

[43] Le Cam L. (1986). Asymptotic Methods in Statistical Decision Theory.

[44] Majtey A.P., Lamberti P.W., Prato D.P. (2005). Jensen-Shannon divergence as a measure of distinguishability between mixed quantum states. Phys. Rev. A.

[45] Osán T.M., Bussandri D., Lamberti P. (2018). Monoparametric family of metrics derived from classical Jensen–Shannon divergence. Phys. A Stat. Mech. Its Appl..

[46] Briët J., Harremoës P. (2009). Properties of classical and quantum Jensen-Shannon divergence. Phys. Rev. A.

[47] Fuglede B., Topsoe F. (2004). Jensen-Shannon divergence and Hilbert space embedding. Proceedings of the International Symposium onInformation Theory, ISIT 2004 Proceedings.

[48] Endres D.M., Schindelin J.E. (2003). A new metric for probability distributions. IEEE Trans. Inf. Theory.

[49] Liang Y.C., Yeh Y.H., Mendonça P.E., Teh R.Y., Reid M.D., Drummond P.D. (2019). Quantum fidelity measures for mixed states. Rep. Prog. Phys..

[50] Muthuganesan R., Sankaranarayanan R. (2017). Fidelity based measurement induced nonlocality. Phys. Lett. A.

[51] Liu L., Hou J., Qi X. (2019). Quantum correlation based on Uhlmann Fidelity for Gaussian states. Entropy.

[52] Guo Y., Zhang L., Yuan H. (2020). Entanglement measures induced by fidelity-based distances. Quantum Inf. Process..

[53] Grace M. (2010). Environment-invariant measure of distance between evolutions of an open quantum system. New J. Phys..

[54] Paunković N., Sacramento P., Nogueira P., Vieira V., Dugaev V. (2008). Fidelity between partial states as a signature of quantum phase transitions. Phys. Rev. A.

[55] Shao L.H., Xi Z., Fan H., Li Y. (2015). Fidelity and trace-norm distances for quantifying coherence. Phys. Rev. A.

[56] Schoenberg I.J. (1938). Metric spaces and positive definite functions. Trans. Am. Math. Soc..

[57] Berg C., Christensen J., Ressel P. (1984). Harmonic Analysis on Semigroups.

